# Chinese herbal medicines for mild cognitive impairment: A protocol for meta-analysis and trial sequential analysis

**DOI:** 10.1097/MD.0000000000027323

**Published:** 2021-10-01

**Authors:** Si-Chun Gu, Li-Min Zhang, Chun-Xu Wang, Yan-Jie Qu, Jing-Yi Ma, Rong-Rong Zhen, Chao Gu, Can-Xing Yuan

**Affiliations:** Department of Neurology, Longhua Hospital, Shanghai University of Traditional Chinese Medicine, 725 South Wanping Road, Shanghai, China.

**Keywords:** Chinese herbal medicines, meta-analysis, mild cognitive impairment, protocol, trial sequential analysis

## Abstract

**Background:**

Mild cognitive impairment (MCI), as a common neurodegenerative aging disease representing an intermediate stage between normal cognitive functioning and dementia, poses an excessive burden on health care. The clinical benefit of Chinese herbal medicines (CHMs) for MCI remains inconclusive. This study is aimed at evaluating the efficacy and acceptability of CHMs through meta-analysis and trial sequential analysis (TSA).

**Methods:**

We applied extensive strategies on preliminary literature screening to identify relevant randomized controlled trials which meticulously compare any of CHMs interventions with placebo groups as monotherapy for MCI. The primary outcome of this study is the change of global cognitive function, and the secondary outcomes include assessments of activities of daily living, mood, and adverse events. Data synthesis, risk of bias assessment, sensitivity and subgroup analyses, and TSA will be conducted with application of Review Manager, Stata, and TSA software. The quality of the evidence will be evaluated using the Grading of Recommendations Assessment, Development and Evaluation instrument. INPLASY registration number: INPLASY202190006 (https://inplasy.com/inplasy-2021-9-0006/).

**Results:**

This study will confirm the clinical efficacy and safety of CHMs when used in the treatment of patients with MCI.

**Conclusion:**

This study will provide reliable evidence and references for the selection of CHMs in therapy and future clinical research of MCI.

## 1 Introduction

Mild cognitive impairment (MCI) is the transitional phase between normal cognitive functioning and dementia, characterized by an objective cognitive deficit which has not yet crossed the threshold for dementia, though without notably interference in daily life activities.^[1–3]^ Due to the aging population, the number of people with MCI is expected to grow. The prevalence is estimated to rise to over 2 billion people and by 10% to 20% in the near future among ages over 60.^[4]^ Moreover, individuals with MCI have an increased risk of developing Alzheimer's disease or other dementia. The annual conversion rate from MCI to dementia ranges from 5% to 20%, depending on the sample studied and the follow-up duration.^[5,6]^ These rapidly growing cases and relatively high conversion rate will have a large impact in the families and society, placing an excessive burden on health care. Therefore, the World Health Organization stresses to take global action against MCI, which has long made the development of effective therapies for MCI a critical issue.^[7,8]^

Thus far, there are no recommended pharmacological or non-pharmacological treatments for MCI.^[9]^ Recent meta-analyses show that cholinesterase inhibitors, including donepezil, galantamine, and rivastigmine, have negligible effects in reductions in conversion to dementia or well-known cognitive test scores such as Mini-Mental State Examination (MMSE) and Alzheimer's Disease Assessment Scale–cognitive section (ADAS-cog), and Montreal Cognitive Assessment (MoCA) when prescribed to patients with MCI.^[10,11]^ On the other hand, cholinesterase inhibitors were associated with a higher incidence of adverse events, including abnormal dreams, insomnia, headache, dizziness, nausea, diarrhea, vomiting, and weight loss.^[12,13]^ As for non-pharmacological treatments, there remains uncertainty over the effects of physical exercise, cognitive stimulation, cognitive training, cognitive rehabilitation, musical therapy, and multi-domain interventions in MCI given large diversity existing in different study designs and potential risk of bias in this field.^[14,15]^

**Figure 1:**
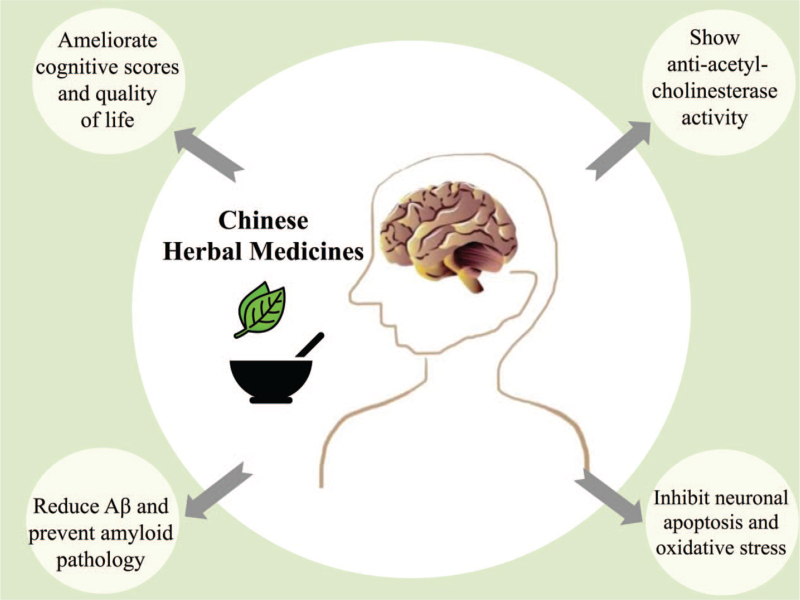
The effects of Chinese herbal medicines with regards to the improvement of mild cognitive impairment.

In this situation, Chinese herbal medicines (CHMs), applied widely to treat MCI in hospitals in China for decades, have gained significant research attention from the global medical community due to their potential as novel treatments for MCI.^[16,17]^ Clinical research has shown that CHMs can ameliorate cognitive function and quality of life with improvement of MMSE, ADAS-cog, MoCA, and Activities of Daily Living (ADL) scores. The results of experimental studies have suggested that CHMs can exert multiple effects, with limited side effects and can act through multiple targets and multiple pathways.^[18,19]^ Compounds found in CHMs have been reported to be able to inhibit the generation of Aβ and prevent amyloid pathology, including ginsenoside, berberine, baicalein, salvianolic acid.^[20,21]^ Some studies on CHMs focusing on the Acetylcholine system have found that Huperzine A, ginsenosides, curcumin, icariin, and ganoderma can exhibit robust anti-acetyl-cholinesterase activity.^[22,23]^ Apart from these, research has also indicated that CHMs can inhibit neuronal apoptosis, stimulate neuronal mitochondrial function and ameliorate oxidative stress in the hippocampus, and so on (Fig. 1).^[24,25]^

Thus, a quantitative meta-analysis including the most recent studies is needed to clarify the efficacy of CHMs for MCI. The primary objective of this meta-analysis is to quantify the overall effect of CHMs on global cognitive function in patients diagnosed with MCI. Secondary objectives are to

1. determine whether CHMs positively influence ADL of MCI patients,
2. evaluate the efficacy of CHMs on mood, and
3. assess the safety and tolerability of the CHMs based on reported adverse events and dropouts.

## 2 Methods

### 2.1 Protocol and registration

**Figure 2:**
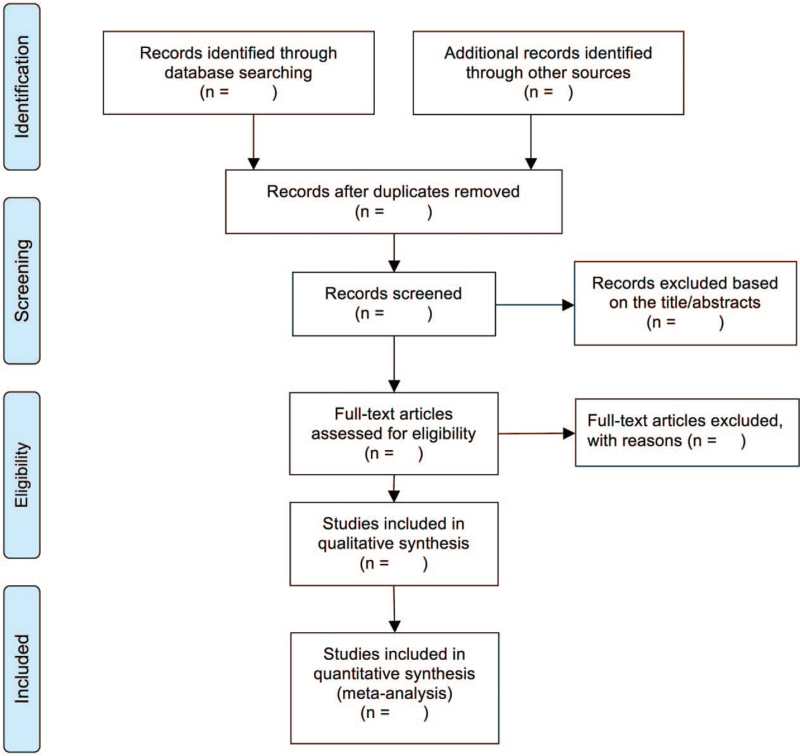
Flow chart of literature selection process.

The current study will be conducted in accordance with the recommendations of the Cochrane Handbook for Systematic Reviews of Interventions and will be reported in compliance with the Preferred Reporting Items for Systematic Reviews and Meta-Analyzes guidelines, as shown in Figure 2.^[26,27]^ All analyzes will be based on the basis of previous published studies and therefore there exists no ethical approval or patient consent are requirements. The protocol has been registered on INPLASY (International Platform of Registered Systematic Review and Meta-analysis Protocols) platform (https://inplasy.com/), registration number: INPLASY202190006 (https://inplasy.com/inplasy-2021-9-0006/).

### 2.2 Literature search

We will apply extensive strategies for preliminary literature search in PubMed, Embase, Cochrane Library, China National Knowledge Infrastructure, China Biological Medicine, Chinese Science and Technology Periodical Database, and Chinese Medicine Premier databases to identify relevant articles with no limits on publication date. Through both combined Medical subject headings and text-terms followed by Boolean logical operators, an exhaustively search was executed by using the following Medical subject headings terms with the limitation of Chinese and English language: “Cognitive Dysfunctions,” “Mild cognitive impairment,” “Cognitive impairment,” “Mild Neurocognitive Disorder,” “Cognitive Decline,” “Randomized controlled trials,” “Drugs, Chinese Herbal,” as well as additional relevant conceptual keywords. References of studies with potential relevance will be manually checked in case of missing a few underlying eligible articles. We will also search www.clinicaltrials.gov and screened the bibliographies to identify any potentially eligible studies.

### 2.3 Selection criteria

The participant (P), intervention (I), comparator (C), outcome (O), and study design (S) are the key factors determining the selection criteria of studies in our analysis.

#### 2.3.1 Type of participant

Participants diagnosed with MCI by any proper clinical criteria not caused by traumatic brain injury or space-occupying lesion will be included. There is no restriction on age, sex, race, or region of the enrolled participants.

#### 2.3.2 Type of interventions and comparators

Interventions in the treatment group will include any kinds of orally administered CHMs such as prescription and Chinese patent medicines in the form of liquids, pills, powders, granules, or capsules as mono-therapy. The control group was defined as patients who were assigned to receive placebo. CHMs in combination with other conservative treatments such as cognitive rehabilitation and oral drugs, and other Traditional Chinese medicine (TCM) treatments such as intravenous medication, acupuncture, and massage for cognitive deficit will be excluded. The concomitant use of medication for noncognitive disorders will be allowed.

#### 2.3.3 Type of outcomes

The change in global cognitive function scores will be applied as the primary outcome measure, derived from MMSE, MoCA, and ADAS-cog scales.^[28–30]^ Secondary outcomes include assessments of ADL and mood which might be measured by the Bayer ADL, Erlangen ADL, Alzheimer Disease Cooperative Study ADL, the Disability Assessment for dementia ADL scales, and Geriatric Depression Scale.^[31–35]^ The incidences of adverse events related to CHMs will also be included as secondary outcomes. All outcome measures have to be administered at baseline and directly after the intervention period.

#### 2.3.4 Study design

All relevant peer-reviewed articles with a randomized controlled trial design will be included. Case reports, reviews, prospective or retrospective cohort studies, conference abstracts, and studies not written in English or Chinese will be excluded. When articles reported an overlap in the sample of participants the article with the largest sample was included.

### 2.4 Literature selection and data extraction

Two researchers (S.C.G. and L.M.Z.) will independently check the titles and abstracts of literatures according to inclusion and exclusion criteria, and identify relevant records as included, excluded or uncertain. In case of uncertainty, the full-text article was acquired to identify eligibility. Disagreements were discussed with other researchers (C.X.W. and C.G.) and adjusted after reaching consensus. The Endnote X7 literature management software (Thompson ISI Research Soft, Philadelphia, Pennsylvania, USA) will be used for procession of the screening records. Data extraction will be performed by another 2 researchers (Y.J.Q. and J.Y.M.) independently from the primary texts, supplementary appendixes and protocols. Collected features include title, year of publication, author, recruitment period, country of centers, sample size of each group, details regarding trial design (e.g., randomization, allocation concealment, blinding, etc.), patient demographics, treatment and control interventions, and outcomes data. Disagreements will be resolved by consensus.

### 2.5 Risk of bias assessment

Three researchers (S.C.G., L.M.Z. and C.X.Y.) will independently assess risk of bias with use of the Cochrane Collaboration's tool.^[36]^ Risk of bias at trial level is reported in 6 domains:

1. selection bias (random sequence generation and allocation concealment);
2. performance bias (blinding of participants and personnel);
3. detection bias (blinding of outcome assessment);
4. attrition bias (incomplete outcome data);
5. reporting bias (selective reporting); and
6. other bias including financial and academic bias.

Each domain will be rated as low, high, or unclear, and a total risk of bias judgment is based on the assessment of all domains. Trials with low risk of bias for all key domains are judged as low risk of bias. Trials with low risk of bias in all domains except blinding of participants and personnel are adjudicated as overall intermediate risk of bias. Trials with high risk of bias for greater than or equal to one key domain are judged as at high risk of bias. We plan to base our main1 conclusions on the results from trials with overall low or intermediate risk of bias in case no trials were adjudicated as having overall low risk of bias. Differences will be discussed with a third researcher (R.R.Z.) until consensus is reached.

### 2.6 Statistical analysis

#### 2.6.1 Measures of treatment effect

We will report continuous outcomes as mean differences with standard deviations and dichotomous outcomes as relative risks with 95% confidence intervals (CIs). The primary outcome of the change in global cognitive function scores will be treated as continuous variables and mean differences with 95% CIs between treatment and control groups will be estimated. Intention to treat analyses will be reported if available. All p values are two-tailed and considered statistically significant if less than 0.05.

#### 2.6.2 Heterogeneity test

Statistical heterogeneity between the trials will be assessed primarily by inspecting Forest plots for heterogeneity, secondarily using the inconsistency (*I*^2^) and diversity (D^2^) statistics with thresholds as suggested by the Cochrane Handbook, with *I*^2^ values of 0% to 25%, 25% to 75%, and 75% to 100% representing low, moderate, and substantial heterogeneity.^[37]^ We will use random-effects models (assuming that the true intervention effects in the included trials are not identical but follow normal distribution) or fixed-effects models (assuming that the true effect of the intervention in both direction and magnitude is fixed across included trials) across outcomes according to the heterogeneity and report the most conservative estimates with the widest CIs. Fixed-effects models will be applied if there is no evidence of heterogeneity; otherwise, random-effects models will be applied.

#### 2.6.3 Sensitivity analysis

If necessary, a leave-one-out sensitivity analysis will be performed to evaluate the main trials demonstrating a substantial impact on the inter-study heterogeneity. If there is no qualitative change in the combined effect, the results are stable.

#### 2.6.4 Subgroup analysis

We plan to conduct 5 subgroup analyses of the included trials to evaluate the influences of the following factors on primary outcome:

1. patient age;
2. type of CHM;
3. MCI duration;
4. sample size;
5. cognitive function scores at the baseline.

Heterogeneity in the subgroup analyses is assessed using the Chi^2^ test with *P* = .05 considered significant. And only subgroup analyzes showing a statistically significant test of interaction (*P* < .05) will be considered to provide evidence of an intervention effect.

#### 2.6.5 Publication bias

Publication bias will be assessed by visual inspection of the funnel plots generated by Review Manager software (version 5.3, Cochrane Collaboration, Oxford, UK), which display the relationship between effect size and sample size. Trials distributed symmetrically around the mean effect size in a funnel shape indicates that there is no publication bias. If the plot is asymmetric and there is no inverted funnel shape, it indicates that there might be publication bias and trials falling outside the funnel shape have high risk of bias. Publication bias will also be quantified by the Begg-Mazumdar test^[38]^ and the Egger test using Stata software (version 15.1, Stata Corp, College Station, TX).^[39]^

#### 2.6.6 Grading quality of evidence

Two authors independently evaluated the quality assessment of the selected studies according to the Grading of Recommendations Assessment, Development and Evaluation (GRADE) instrument.^[40]^ The overall quality of evidence was rated “high,” “moderate,” “low” or “very low” based on our evaluation of identified risks of bias, inconsistency, indirectness, imprecision, and publication bias. And the summary table will be constructed with the GRADE Profiler (version 3.6, GRADEpro).

#### 2.6.7 Dealing with missing data

The corresponding authors will be contacted regarding missing data at least twice. We plan to conduct sensitivity analyses for participants who are lost to follow-up with best-worst- and worst-best-case-scenarios.

### 2.7 Trial sequential analysis

Cumulative meta-analyses may result in type I errors with the increased risk of random errors due to repetitive statistical testing especially when trials included have a small sample size, publication bias, low quality.^[41]^ Trial sequential analysis (TSA) is a method aiming to correct for the risk of random errors. For determining the effect while adjusting the threshold for statistical significance, the optimal information size (IS) is quantified for each variable based on a value of 0.05 for α (type I error of 5%, two-sided) and 0.20 for β (80% power): for continuous data, the required IS is estimated based on diversity-adjusted IS as 50%, MD, and variance based on empirical assumptions, whereas, for dichotomous data, the required IS is based on the incidence of low risk of bias studies, which are auto-generated by the software. TSA also creates trial sequential monitoring boundaries, futility boundaries, and areas for benefit, harm, and futility simultaneously to eliminate early positive findings and reach more reliable conclusions. If the cumulative z curve exceeds the IS, or crosses the trial sequential monitoring boundary or enters the futility area, firm evidence may have been made from the difference between the 2 interventions (CHM vs pyrrolidinone derivatives) and no further studies are needed.^[42]^ Otherwise, there is insufficient evidence to reach a conclusion. In the present analysis, we will perform a TSA to assess the impact of random error and repetitive testing for the primary outcome using TSA software (version 0.9.5.10 Beta, Copenhagen Trial Unit, Denmark; http://www.ctu.dk/tsa).

## 3 Discussion

Due to the substantial-dual harm caused by MCI in the families and society, it is imperative to provide a reliable evidence of positively affecting global cognitive function, ADL and mood in MCI patients with by enhancing relevant cognitive interventions with good clinical tolerance.

In recent years, in contrast to the slight efficacy of the western medicine in the treatment of MCI, CHMs have shown remarkable efficacy in terms of improving MCI. In addition to above-mentioned incentive mechanisms of CHMs, the independent TCM theory system of MCI contained in CHMs has been attracting a great deal of attention in this field. TCM believes that the pathogenic process of MCI involves a deficiency of qi, blood, yin and yang, phlegm, and stagnation, causing the brain to lose nourishment, and thus undergo atrophy, thus leading to amnesia and retard. Although the brain is the organ involved, the disease also involves the kidney, heart, liver, and spleen. The nature of this disease is principally deficiency and secondary excess.

Although the current understanding of the theoretical, clinical and experimental research has led to the realization that CHMs provide significant potential for development as promising therapeutics for MCI, the evidence might be limited when the evaluation included comparison with active control groups or incorporated more than 2 cognitive treatments simultaneously. Therefore, as the first meta-analysis of CHMs as mono-therapy compared with placebo for MCI, our study attempts to summarize and estimate the most recent studies to provide a robust and conclusive evidence for the improvement of MCI by CHMs with the application of TSA.

Several limitations may exist in this study. First, since MCI has neuropathological heterogeneity, randomized controlled trial conducted for neuropathologically classified MCI with specific biomarker data are rare. Thus, the relationship between the efficacy of CHM and neuropathological features of MCI may not be discovered. Second, there might be the paucity of studies included for the individual CHM. Owing to the different pharmacological mechanisms of action, studies regarding the effectiveness of individual CHM for MCI are needed in the further. Third, the rate of conversion from MCI to dementia is about 10% each year and the pace of cognitive decline for the individual patient with MCI is quite slow, which might result in the relatively short study duration included in our meta-analysis. Though our subgroup analysis of MCI duration may contribute to show the correlation between the duration and cognitive function scores, studies with longer duration are still needed to determine the overall benefits of CHM for MCI.

## Acknowledgments

We acknowledge all the authors for their helpful suggestions in this study during the COVID-19 outbreak.

## Author contributions

**Conceptualization:** Si-Chun Gu, Chao Gu, Can-Xing Yuan.

**Data curation:** Si-Chun Gu, Li-Min Zhang, Chun-Xu Wang, Yan-Jie Qu, Jing-Yi Ma, Rong-Rong Zhen.

**Formal analysis:** Si-Chun Gu, Li-Min Zhang.

**Funding acquisition:** Si-Chun Gu, Li-Min Zhang.

**Investigation:** Si-Chun Gu, Li-Min Zhang.

**Methodology:** Si-Chun Gu, Li-Min Zhang.

**Project administration:** Si-Chun Gu, Li-Min Zhang.

**Resources:** Si-Chun Gu, Li-Min Zhang.

**Software:** Si-Chun Gu, Li-Min Zhang.

**Supervision:** Chao Gu and Can-Xing Yuan.

**Validation:** Can-Xing Yuan.

**Writing – original draft:** Si-Chun Gu, Li-Min Zhang.

**Writing – review & editing:** Chao Gu, Can-Xing Yuan.

## References

[1] Langa KM Levine DA. The diagnosis and management of mild cognitive impairment: a clinical review. JAMA 2014;312:2551–61. 25514304 10.1001/jama.2014.13806 PMC4269302

[2] Petersen RC Smith GE Waring SC. Mild cognitive impairment: clinical characterization and outcome. Arch Neurol 1999;56:303–8. 10190820 10.1001/archneur.56.3.303

[3] Petersen RC. Clinical practice. Mild cognitive impairment. N Engl J Med 2011;364:2227–34. 21651394 10.1056/NEJMcp0910237

[4] Plassman BL Langa KM Fisher GG. Prevalence of cognitive impairment without dementia in the United States. Ann Intern Med 2008;148:427–34. 18347351 10.7326/0003-4819-148-6-200803180-00005 PMC2670458

[5] Roberts RO Knopman DS Mielke MM. Higher risk of progression to dementia in mild cognitive impairment cases who revert to normal. Neurology 2016;82:317–25. 10.1212/WNL.0000000000000055 PMC3929198 24353333

[6] Sarah Tomaszewski F Dan M Reed BR. Progression of mild cognitive impairment to dementia in clinic- vs community-based cohorts. Arch Neurol 2009;66:1151–7. 19752306 10.1001/archneurol.2009.106 PMC2863139

[7] Nakanishi M Nakashima T. Features of the Japanese national dementia strategy in comparison with international dementia policies: How should a national dementia policy interact with the public health- and social-care systems? Alzheimer Dement 2014;10:468–76. 10.1016/j.jalz.2013.06.005 23954026

[8] Petersen RC. Mild cognitive impairment as a clinical entity and treatment target. Arch Neurol 2004;62:1160–3. discussion 1167. 10.1001/archneur.62.7.1160 16009779

[9] Tricco AC Soobiah C Berliner S. Efficacy and safety of cognitive enhancers for patients with mild cognitive impairment: a systematic review and meta-analysis. CMAJ 2013;185:1393–401. 24043661 10.1503/cmaj.130451 PMC3826344

[10] Hansen RA Gartlehner G Webb AP. Efficacy and safety of donepezil, galantamine, and rivastigmine for the treatment of Alzheimer's disease: a systematic review and meta-analysis. Clin Intervent Aging 2008;3:211–25. PMC2546466 18686744

[11] Di Santo SG Prinelli F Adorni F. A meta-analysis of the efficacy of donepezil, rivastigmine, galantamine, and memantine in relation to severity of Alzheimer's disease. J Alzheimers Dis 2013;35:349–61. 23411693 10.3233/JAD-122140

[12] Anderson ND. State of the science on mild cognitive impairment (MCI). CNS Spectr 2019;24:78–87. 30651152 10.1017/S1092852918001347

[13] Matsunaga S Fujishiro H Takechi H. Efficacy and safety of cholinesterase inhibitors for mild cognitive impairment: a systematic review and meta-analysis. J Alzheimers Dis 2019;71:513–23. 31424411 10.3233/JAD-190546

[14] Blondell SJ Hammersley-Mather R Veerman JL. Does physical activity prevent cognitive decline and dementia? A systematic review and meta-analysis of longitudinal studies. BMC Public Health 2014;14:01–12. 10.1186/1471-2458-14-510 PMC4064273 24885250

[15] Gates N Fiatarone Singh MA Sachdev PS. The effect of exercise training on cognitive function in older adults with mild cognitive impairment: a meta-analysis of randomized controlled trials. Am J Geriatr Psychiatry 2013;21:1086–97. 23831175 10.1016/j.jagp.2013.02.018

[16] Yan B Wang J Xue Z. Chinese Medicinal Herbs in the Treatment of Diabetic Cognitive Impairment: A Systematic Review and Meta-Analysis. Evid Based Complement Alternat Med 2018;2018:7541406–16. 30302118 10.1155/2018/7541406 PMC6158952

[17] Dey A Bhattacharya R Mukherjee A. Natural products against Alzheimer's disease: Pharmaco-therapeutics and biotechnological interventions. Biotechnol Adv 2017;35:178–216. 28043897 10.1016/j.biotechadv.2016.12.005

[18] Dong L May BH Feng M. Chinese herbal medicine for mild cognitive impairment: a systematic review and meta-analysis of cognitive outcomes. Phytother Res 2016;30:1592–604. 27416935 10.1002/ptr.5679

[19] Ji HF Shen L. Berberine: a potential multipotent natural product to combat Alzheimer's disease. Molecules 2011;16:6732–40. 21829148 10.3390/molecules16086732 PMC6264702

[20] Jia L Liu J Song Z. Berberine suppresses amyloid-beta-induced inflammatory response in microglia by inhibiting nuclear factor-kappa B and mitogen-activated protein kinase signalling pathways. J Pharm Pharmacol 2012;64:1510–21. 22943182 10.1111/j.2042-7158.2012.01529.x

[21] Cai GL Fang S Zhang QH. Advances in the pharmaceutical research of shizhen snakefoot. Res Develop Nat Prod 2015;27:931–9.

[22] Yang G Wang Y Tian J. Huperzine A for Alzheimer's disease: a systematic review and meta-analysis of randomized clinical trials. PLoS One 2013;8:e74916–24. 24086396 10.1371/journal.pone.0074916 PMC3781107

[23] He Z Pan Z Lu W. Neuroprotective effects of tanshinone II A on vascular dementia in rats. Chin J Chin Mater Med 2010;14:1883–6. 10.4268/cjcmm20101426 20939291

[24] Cui J Wang J Zheng M. Ginsenoside Rg2 protects PC12 cells against (-amyloid25-35-induced apoptosis via the phosphoinositide 3-kinase/Akt pathway. Chem Biol Interact 2017;275:152–61. 28756148 10.1016/j.cbi.2017.07.021

[25] Gong JW Ye Y Zhang XL. Effects of Dihuang Yinzi on the SOD, CAT, GSH-Px activities and MDA contents in the serum and brain of cerebral ischemia-reperfusion model rats. J Ethnopharmacol 2013;142:754–61.

[26] Cumpston M Li T Page MJ. Updated guidance for trusted systematic reviews: a new edition of the Cochrane Handbook for Systematic Reviews of Interventions. Cochrane Database Syst Rev 2019;10:ED000142-5. 10.1002/14651858.ED000142 PMC10284251 31643080

[27] Moher D Liberati A Tetzlaff J. Preferred reporting items for systematic reviews and meta-analyses: the PRISMA Statement. Open Med 2009;3:e123–30. 21603045 PMC3090117

[28] Folstein MF Folstein SE McHugh PR. “Mini-mental state”. A practical method for grading the cognitive state of 494 patients for the clinician. J Psychiatr Res 1975;12:189–98. 1202204 10.1016/0022-3956(75)90026-6

[29] Nasreddine ZS Phillips NA Bedirian V. The Montreal cognitive assessment, MoCA: A brief 498 screening tool for mild cognitive impairment. J Am Geriatr Soc 2005;53:695–9. 15817019 10.1111/j.1532-5415.2005.53221.x

[30] Rosen WG Mohs RC Davis KL. A new rating scale for Alzheimer's disease. Am J Psychiatry 1984;141:1356–64. 6496779 10.1176/ajp.141.11.1356

[31] Erzigkeit H Lehfeld H Peña-Casanova J. The Bayer-Activities of Daily Living Scale (B-ADL): results from a validation study in three European countries. Dement Geriatr Cogn Disord 2001;12:348–58. 11455136 10.1159/000051280

[32] Graessel E Viegas R Stemmer R Küchly B Kornhuber J Donath C. The Erlangen test of activities of daily living: first results on reliability and validity of a short performance test to measure fundamental activities of daily living in dementia patients. Int Psychogeriatr 2009;21:103–12. 18925975 10.1017/S1041610208007710

[33] Galasko D Bennett D Sano M. An inventory to assess activities of daily living for clinical trials in Alzheimer's disease. The Alzheimer's Disease Cooperative Study. Alzheimer Dis Assoc Disord 1997;11:S33–9. 9236950

[34] Feldman H Sauter A Donald A. The disability assessment for dementia scale: a 12-month study of functional ability in mild to moderate severity Alzheimer disease. Alzheimer Dis Assoc Disord 2001;15:89–95. 11391090 10.1097/00002093-200104000-00008

[35] Yesavage JA Brink TL Rose TL. Development and validation of a geriatric depression screening scale: a preliminary report. J Psychiatr Res 1982–1983;17:37–49. 10.1016/0022-3956(82)90033-4 7183759

[36] Higgins JP Altman DG Gotzsche PC. The Cochrane Collaboration's tool for assessing risk of bias in randomised trials. BMJ 2011;343:d5928-37. 10.1136/bmj.d5928 PMC3196245 22008217

[37] Higgins JP Thompson SG. Quantifying heterogeneity in a meta-analysis. Stat Med 2002;21:1539–58. 12111919 10.1002/sim.1186

[38] Begg CB Mazumdar M. Operating characteristics of a rank correlation test for publication bias. Biometrics 1994;50:1088–101. 7786990

[39] Egger M Davey Smith G Schneider M. Bias in meta-analysis detected by a simple, graphical test. BMJ 1997;315:629–34. 9310563 10.1136/bmj.315.7109.629 PMC2127453

[40] Guyatt GH Oxman AD Vist GE. GRADE: an emerging consensus on rating quality of evidence and strength of recommendations. BMJ 2008;336:924–6. 18436948 10.1136/bmj.39489.470347.AD PMC2335261

[41] Wang B Du W Jia Y. Cytotoxic T-lymphocyte-associated protein 4+49A/G polymorphisms contribute to the risk of type 1 diabetes in children: An updated systematic review and meta-analysis with trial sequential analysis. Oncotarget 2017;8:1053–6. 10.18632/oncotarget.14457 PMC5354680 28060767

[42] Thorlund K Engstrøm J Wetterslev J. User Manual for Trial Sequential Analysis (TSA). 2011;Copenhagen, Denmark: Copenhagen Trial Unit, Centre for Clinical Intervention Research, 1–115.

